# Variables Influencing Cryptocurrency Use: A Technology Acceptance Model in Spain

**DOI:** 10.3389/fpsyg.2019.00475

**Published:** 2019-03-18

**Authors:** Mario Arias-Oliva, Jorge Pelegrín-Borondo, Gustavo Matías-Clavero

**Affiliations:** ^1^Department of Business Management, Rovira i Virgili University, Tarragona, Spain; ^2^Economy and Business Department, University of La Rioja, Logroño, Spain; ^3^Economic Structure and Development Economics, Autonomous University of Madrid, Madrid, Spain

**Keywords:** cryptocurrencies, bitcoin, blockchain, ICO, initial coin offering, fintech

## Abstract

The first commercial transaction with the first cryptocurrency in 2010 marked the start of a revolution in transactions. Blockchain and cryptocurrencies will dramatically transform how we do transactions, just as the Internet revolutionized how we communicate. Currently, more than 2,000 cryptocurrencies are quoted on the market, and many more are being launched in initial coin offerings for use as an exchange method in a specific business ecosystem or as rights to assets or liabilities. As an emerging fintech, cryptocurrencies open up many opportunities, but they also pose significant challenges and limitations. This paper analyzes the key factors for the successful development of a cryptocurrency from a consumer-behavior perspective. Using a technology acceptance theoretical framework, we test a model able to explain almost 85% of the intention to use cryptocurrencies. Surprisingly, risk was not a significant factor. This could be because most of the respondents considered operating with cryptocurrencies to be risky; the lack of variability in their responses to the questions about perceived risk would explain this lack of explanatory power. However, willingness to manage cryptocurrency risk could be a precondition for adoption. The performance expectancy for a given cryptocurrency was the most important factor for its success. The research was conducted in Spain with college-educated adults with basic knowledge of the Internet.

## Introduction

The origin of blockchain and cryptocurrencies dates back to 2008, when Satoshi Nakamoto – the pseudonymous developer of blockchain and the cryptocurrency bitcoin – posted a paper to a cryptography forum entitled “Bitcoin: A Peer-to-Peer Electronic Cash System” (Nakamoto, 2008a; Simonite, 2011). The paper described a revolutionary technology to create a genuine decentralized peer-to-peer monetary system, arguing that “a purely peer-to-peer version of electronic cash would allow online payments to be sent directly from one party to another without going through a financial institution” (Nakamoto, 2008b, p. 1). Blockchain is defined as “a digital, distributed transaction ledger, with identical copies maintained on multiple computer systems controlled by different entities” (Schatsky and Muraskin, 2015, p. 2). Cryptocurrencies are based on blockchain but are not the only possible application. There is a dangerous relationship between blockchain and cryptocurrencies (Carson et al., 2018), being necessary to underline that cryptocurrencies are one of the multiple possibilities of blockchain technologies. According to the World Economic Forum (2015) 10% of GDP will be stored in blockchain by 2027 (World Economic Forum, 2015), with an average annual growth rate of 62.1% until 2025 (Business Wire, 2017).

Although blockchain is expected to dramatically impact and have applications in most economic sectors and activities, at present cryptocurrencies remain more important. The World Bank defines a non-fiat digital currency as a digital currency that is not backed by any underlying asset, has zero intrinsic value, and does not represent a liability on any institution (Natarajan et al., 2017). Digital currencies based on blockchain technology, which employs cryptographic techniques, are considered cryptocurrencies. The U.S. Federal Reserve considers the current payment system to be slow, insecure, inefficient, uncollaborative, and non-global (Federal Reserve System, 2017). Cryptocurrencies are seen as a potential instrument for solving all these problems (Deloitte, 2015).

From the start of this revolution with the launch of bitcoin, the first cryptocurrency, the business and economic worlds have sought to adapt and integrate the new financial technology into their activities. In 2010, the first retail purchase was made with Bitcoins. Laszlo Hanyecz paid 10,000 bitcoins for two pizzas (Bort, 2014). Today, you can hire a lawyer, buy a car, or pay for a doctor’s appointment with bitcoins at 5,040 businesses around the world (Coinmap, 2018; Usebitcoins, 2018). But bitcoin is only one of 2,094 cryptocurrencies on the market (Coin Market Cap, 2018), which range from bitcoin itself, still the most well-known with a market capitalization over US$110 billion, to largely unknown cryptocurrencies launched more recently, such as Harmonycoin, with a capitalization of just US$107 (Coin Market Cap, 2018). The volatility of cryptocurrencies opens enormous psychological thresholds in prices (Pelegrín-Borondo et al., 2015). Nor does that number include all cryptocurrencies, just the ones quoted on the market to be bought and sold. Today, any business can create its own cryptocurrency using blockchain technology and determine its use through an initial coin offering (ICO). The new cryptocurrency can be used as an internal business ecosystem payment method to grant access to the products or services the ecosystem offers; it can represent a right to an asset or liability; or it can be used as a speculative cryptocurrency whose value is based on market expectations. The range is very wide and will only grow wider in the coming years. For example, according to the October report by the ICO rating platform ICObench (2018). which analyzes part of all global ICOs that are launched, from October 8, 2018, to October 14, 25 new ICOs were begun, 557 were ongoing, and 23 were completed, raising US$87,396,196 in funds (ICObench, 2018). A 2017 survey of 902 tracked ICOs showed that 59% were considered totally or partially failed with a total funding of US$233 million (Morris, 2018).

All of this raises the question: what are the key factors that cause a cryptocurrency to be accepted by consumers and/or investors?

As noted, this “cryptocurrency chaos” poses many opportunities, but also many problems. Illegal activities with cryptocurrencies are a fact, especially with bitcoin, the first and most frequently used (Turner et al., 2018). For instance, cryptocurrencies have been used for tax evasion, money laundering, contraband transactions, extortion, and the theft of bitcoins themselves (Bloomberg, 2017). Another drawback is that cryptocurrencies are not an easy technology to use; operating with bitcoins is a major challenge for many users (Krombholz et al., 2017). One qualitative study found that non-users of bitcoin felt incapable of using it Gao et al. (2016), indicating a barrier to the widespread use of cryptocurrencies. In addition to the lack of technological know-how, financial literacy can also constrain the development of cryptocurrencies. In a 2015 financial capability study conducted in the United States, the percentage of respondents capable of correctly answering at least 4 of 5 basic financial literacy questions on a financial literacy test (basic calculations and questions about interest rates, inflation, bond prices, mortgages, and risk) was 37% (Lin et al., 2016). Given this low level of financial literacy, explaining financial concepts related to cryptocurrencies could be difficult (CCN, 2016). Social perception will also be key to cryptocurrency development. An ING study of opinions about bitcoin found that 29% of Europeans would never invest in cryptocurrencies, perceiving shares as a less risky investment tool (Exton and Doidge, 2018).

In short, cryptocurrencies open up many opportunities, such as fast, efficient, traceable, and secure transactions, but also have drawbacks, such as their inherent risk, the technological and financial difficulty of using them, and the uncertain social perception of owning them. The complexity and consequences of the blockchain and cryptocurrency revolution make it imperative to analyze its impacts and challenges from an interdisciplinary perspective. Although some research has been done on bitcoin, as the most widely used and important cryptocurrency today (Holub and Johnson, 2018), the literature on cryptocurrencies in general is scarce, mainly due to their novelty. This paper focuses on the critical factors that any cryptocurrency must consider to succeed in the emerging and chaotic cryptocurrency market. Specifically, it uses technology acceptance models to analyze the influence of perceived risk, performance expectancy, facilitating conditions, effort expectancy, social influence, and financial literacy on the intention to use cryptocurrencies. Determining the key factors for customer acceptance of cryptocurrencies would let current and future market players focus on the most important features a cryptocurrency should have. The research was conducted in Spain with a sample of college-educated adults with basic knowledge of the Internet.

## Literature Review

The Unified Theory of Acceptance and Use of Technology (UTAUT) (Venkatesh et al., 2003) and its extension UTAUT2 (Venkatesh et al., 2012) are models to explain how an emerging technology is accepted by people and organizations. Both are based on Technology Acceptance Models (TAM and TAM2) (Davis, 1989; Venkatesh and Davis, 2000), which, in turn, are rooted in the theory of reasoned action (TRA) (Fishbein and Ajzen, 1975) and the theory of planned behavior (TPB) (Ajzen, 1991). UTAUT models define a direct and positive influence of performance expectancy, social norm, and facilitating conditions on the intention to use a technology.

Performance expectancy is defined as the degree to which a person considers that using a specific technology would be useful to enhance his or her performance. Effort expectancy is defined as the degree of ease associated with the use of a specific technology. Social influence is defined as the degree to which a person perceives that others believe that he or she should use a specific technology. Facilitating conditions are defined as the degree to which a person believes that he or she has the necessary organizational and technical infrastructure to use a specific technology (Venkatesh et al., 2003).

Several studies have looked at the influence of these variables on the acceptance of financial technologies, or fintech, but no consensus has been reached regarding their influence on the intention to use them. On the contrary, important differences have been found depending on the type of technology and target segment. For instance, Moon and Hwang (2018) show that effort expectancy and social influence positively affect the intention to use crowdfunding, but find no evidence that performance expectancy and facilitating conditions do. In contrast, Kim et al. (2018) find that performance expectancy, effort expectancy, and social influence all positively affect the intention to use a payment authentication system based on biometrics. Makanyeza and Mutambayashata (2018) show that while performance expectancy and effort expectancy positively influence the behavioral intention to adopt plastic money, social influence and facilitating conditions do not significantly affect it. Sánchez-Torres et al. (2018) demonstrate that performance expectancy and effort expectancy have a positive impact on the use of financial websites in Colombia. Khan et al. (2017) show that performance expectancy and facilitating conditions are important antecedents of the behavioral intention to use online banking, but find no evidence that effort expectancy and social influence have any significant effect on this intention.

Several studies have likewise looked at the adoption of mobile banking (m-banking). For instance, Farah et al. (2018) determines that performance expectancy, effort expectancy, and social influence are predictors of the intention to use m-banking services in Pakistan, but facilitating conditions have no influence on its adoption. Warsame and Ireri (2018) show that for some consumer segments (based on age, gender, and religion) performance expectancy and effort expectancy significantly influence the intention to use mobile microfinance services, while for others these factors do not affect acceptance. These authors further demonstrate that social influence affects the intention to use mobile microfinance services in all segments. In their study of mobile payment adoption specifically by the base-of the-pyramid (BoP) segment, i.e., people with a very low level of income, Hussain et al. (2018) find that performance expectancy, effort expectancy, facilitating conditions, and social influence all significantly influence behavioral intention. Focusing on m-banking in Bangladesh, Mahfuz et al. (2016) show that effort expectancy and social influence are the most significant antecedents of behavioral intention. Additionally, they find that while performance expectancy and facilitating conditions do not significantly affect the intention to use this technology, facilitating conditions do affect actual use of it. In another study conducted in Bangladesh, Nisha (2016) demonstrates that performance expectancy, effort expectancy, and facilitating conditions significantly influence customers’ intention to use m-banking services. Similarly, in a study conducted in Karnataka, in rural India, Kishore and Sequeira (2016) show that performance expectancy, effort expectancy, and social influence have significant explanatory power with regard to the adoption of m-banking.

As for the literature specifically on cryptocurrencies and bitcoin, Mendoza-Tello et al. (2018) show that perceived usefulness is the most influential factor in the intention to use cryptocurrencies for electronic payments, but find no support for the direct effect of social influence on the intention to use them. According to another study on cryptocurrency adoption based on the TPB, subjective norms (social influence) and perceived behavioral control (how easy or difficult it is to use cryptocurrencies) are significant (Schaupp and Festa, 2018): people who perceive cryptocurrencies as easy to use and people receiving a positive social influence regarding their use are more likely to use them. Bitcoin has also been analyzed as a cryptocurrency. In an acceptance study in China, Shahzad et al. (2018) find that both perceived usefulness and perceived ease of use significantly influence the intention to use bitcoin.

Based on these findings regarding the acceptance of financial technologies, the following hypotheses are proposed:

H1. Performance expectancy regarding the use of cryptocurrencies positively influences the intention to use them.
H2. Effort expectancy regarding the use of cryptocurrencies positively influences the intention to use them.
H3. Social influence regarding the use of cryptocurrencies positively influences the intention to use them.
H4. Facilitating conditions for the use of cryptocurrencies positively influences the intention to use them.

From a behavioral research perspective, Faqih (2016) defines perceived risk as consumers’ perception of the degree of uncertainty and possible undesirable consequences of using or buying a product. Perceived risk has been considered a determinant of consumer behavior in the context of purchase intention (e.g., Salisbury et al., 2001; Kannungo and Jain, 2004), as well as a predictor of technology adoption (e.g., Featherman and Pavlou, 2003). Several recent studies analyze the influence of perceived risk on the intention to use financial technologies with contradictory results. In their study of the intention to use online banking, Khan et al. (2017) validate perceived security as an important antecedent of behavioral intentions. Kishore and Sequeira (2016) show that perceived risk has significant moderate explanatory power with regard to the adoption of m-banking in rural areas. Shaikh et al. (2018) determine that while the direct influence of perceived risk on the intention to use m-banking is generally weak, it plays an important role in the pre-adoption process, influencing other variables that later directly affect the intention to use. Farah et al. (2018) does not find that perceived risk is a determinant variable in the intention to use m-banking in Pakistan. Likewise, Moon and Hwang (2018) find no evidence that perceived risk negatively affects the intention to use crowdfunding.

With regard to the literature on cryptocurrencies in particular, Mendoza-Tello et al. (2018) show that perceived risk is not significant in explaining the intention to use cryptocurrencies for electronic payments.

Based on the understanding of cryptocurrencies as an emerging fintech entailing potential risk, the following hypothesis is proposed:

H5. The perceived risk of using cryptocurrencies negatively influences the intention to use them.

Stolper and Walter (2017) define financial knowledge as the degree of knowledge a person has about key financial concepts and their capacity to apply that knowledge to their financial decision-making.

Several studies demonstrate that financial knowledge is a predictive variable of financial behaviors. Van Rooij et al. (2011) show that financial literacy affects financial decision-making: people with low financial literacy are much less likely to invest in stocks. In their review of the literature on the topic, Lusardi and Mitchell (2014) find that numerous papers demonstrate that the greater a person’s financial knowledge, the more likely he or she is to participate in financial markets and invest in stocks. Their research includes papers from the United States and other countries. Likewise, Stolper and Walter (2017) argue that higher levels of financial knowledge are associated with more saving planning, more saving behavior, more stock market participation, and smarter choices when it comes to the selection of financial products; at the same time, lower levels of financial knowledge are associated with poorer financial decisions, more expensive loans, costly credit card practices, and excessive debt accumulation. In their literature review, Hastings et al. (2013) establish that financial knowledge affects decisions related to the use of credit cards, investments, mortgage loans, and retirement savings plans. Stolper and Walter (2017) report similar findings, showing that many research papers demonstrate that people with a higher level of financial knowledge are more cautious about their financial decisions. Lam and Lam (2017) demonstrate the important influence of financial knowledge on problems related to online shopping, such as addiction or compulsive shopping behaviors.

Given that cryptocurrencies are a technological financial product, and based on the above findings regarding the influence of financial literacy on the use of financial products, the following hypothesis is proposed:

H6. Financial literacy positively influences the intention to use cryptocurrencies.

**FIGURE 1:**
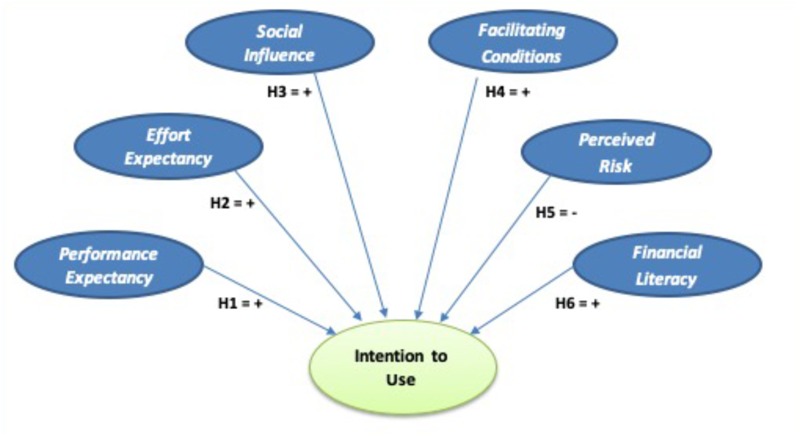
Proposed theoretical model for the intention to use cryptocurrencies.

Figure 1 shows the proposed model for analyzing the intention to use cryptocurrencies.

## Materials and Methods

### Data Collection

We used a structured and self-administered online survey to sample people over the age of 20, living in Spain, who had a university degree. We sent invitations to people with this profile without making any distinctions for age, gender, or household income until we achieved the desired sample size and composition to enable reliable research. Due to the online nature of the survey, the sample is limited to people with a basic command of the Internet.

As noted in the introduction, because cryptocurrencies are based on blockchain technologies, a minimum level of both technological and financial knowledge is needed to have a basic understanding of how to operate with them. Consequently, in order to survey people likely to have a reasonable understanding of these technologies, we focused on college-educated adults. This allowed us to ensure that the respondents would have the minimum required knowledge. This decision regarding the sample was based on other studies that justify the choice of a highly educated sample as a means of making suring that respondents have a higher level of financial knowledge in order to ensure that the collected data will fit the research purpose (Hastings et al., 2013; Lin et al., 2016; Stolper and Walter, 2017).

The sample consisted of 402 people, over the age of 20, living in Spain and with a university degree and a basic grasp of the Internet. The data were collected between August 1 and September 10, 2018.

The survey began with an introductory text about cryptocurrencies and blockchain: “Like knives or fire, new financial technologies have enormous potential, but can be used for good or bad. The innovative blockchain-based financial and insurance services emerging today reduce intermediation and transaction costs, but they could also be insecure and risky if used incorrectly. Cryptocurrencies (such as bitcoin) are a perfect example of blockchain-based financial innovation, offering inalterable, anonymous, and traceable transactions. Today, the technology suffers from significant legal gaps, enabling it to be used for illegal and opaque operations, including tax evasion, money laundering, illegal transactions such as purchasing weapons or drugs, corruption, etc. In addition, it poses other risks, such as the fact that losing your password entails losing your money or that heirs who do not have the key will not be able to access their inheritance.”

With regard to ethics approval: (1) all participants were given detailed written information about the study and procedure; (2) no data directly or indirectly related to the subjects’ health were collected and, thus, the Declaration of Helsinki was not generally mentioned when the subjects were informed; (3) the anonymity of the collected data was ensured at all times; and (4) no permission was obtained from a board or committee ethics approval, it was not required as per applicable institutional and national guidelines and regulations (5) voluntary completion of the questionnaire was taken as consent for the data to be used in research, informed consent of the participants was implied through survey completion.

### Measurement Scales

We based our measurement scales on scales that are widely accepted and used in the literature on technology acceptance. Table 1 shows the constructs, items, and theoretical foundations of each one.

**Table 1:** Constructs, items, and their theoretical foundations.

Construct/item	Theoretical foundation
**Intention to use**	
I intend to use cryptocurrencies	TAM2 scale (Venkatesh and Davis, 2000)
I predict that I will use cryptocurrencies	
**Performance expectancy**	
Using cryptocurrencies will increase opportunities to achieve important goals for me	Adapted from the UTAUT2 scale (Venkatesh et al., 2012)
Using cryptocurrencies will help me achieve my goals more quickly	
Using cryptocurrencies will increase my standard of living	
**Effort expectancy**	
It will be easy for me to learn how to use cryptocurrencies	Adapted from the UTAUT2 scale (Venkatesh et al., 2012)
Using cryptocurrencies will be clear and understandable for me	
It will be easy for me to use cryptocurrencies	
It will be easy for me to become an expert in the use of cryptocurrencies	
**Social influence**	
The people who are important to me will think that I should use cryptocurrencies	Adapted from the UTAUT2 scale (Venkatesh et al., 2012)
The people who influence me will think that I should use cryptocurrencies	
People whose opinions I value would like me to use cryptocurrencies	
**Facilitating conditions**	
I have the necessary resources to use cryptocurrencies	Adapted from the UTAUT2 scale (Venkatesh et al., 2012)
I have the necessary knowledge to use cryptocurrencies	
Cryptocurrencies are compatible with other technologies that I use	
I can get help if I have difficulty using cryptocurrencies	
**Perceived risk**	
Using cryptocurrencies is risky	Faqih (2016) based on Shim and Lee (2011)
There is too much uncertainty associated with the use of cryptocurrencies	
Compared with other currencies/investments, cryptocurrencies are riskier	
**Financial literacy**	
I have a good level of financial knowledge	Based on Hastings et al. (2013)
I have a high capacity to deal with financial matters	

Stolper and Walter (2017) consider that there are two main ways to measure financial literacy: (i) using a test to evaluate a person’s financial knowledge; and (ii) via self-assessments of financial knowledge. We decided to use a self-assessment approach because we consider that people make decisions based on their perception of reality, not reality itself. From a consumer behavior point of view, this means that people will behave according to their perceptions of their financial knowledge, not their actual financial knowledge. The self-conception of financial literacy would thus be the influential factor in relation to the intention to use cryptocurrencies.

### Sample Profile

As already noted, the sample consisted of people over the age of 20, with a university degree and a basic grasp of the Internet. There was a small deviation with regard to gender, with 3% more men than women (53% men). According to López de la Cruz (2002), this is representative of the Spanish population due to women’s later incorporation into higher education.

**FIGURE 2:**
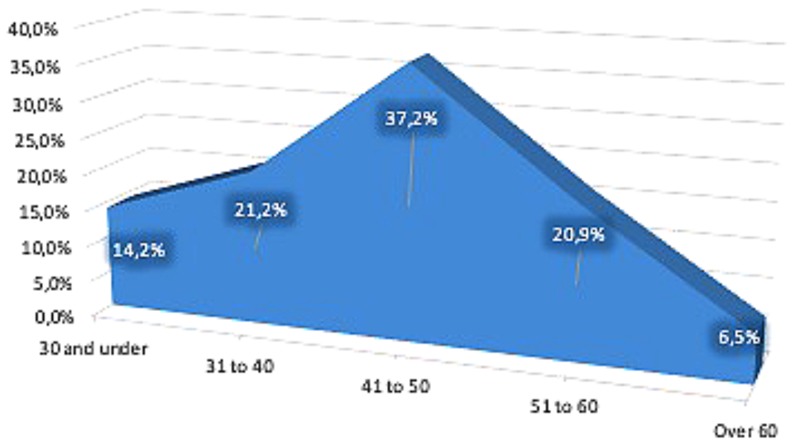
Sample distribution by age.

The sample’s age composition is proportional to the age distribution of the Spanish population at large. Figure 2, 3 show the sample’s age distribution and the Spanish population’s age distribution pyramid. People under the age of 21 were not included because of the very high unlikelihood that they would already have a university degree. The largest segment of respondents was people between the ages of 41 and 50. This is similar to the distribution of the Spanish population as a whole. Therefore, we believe the sample is adequate and representative of the population.

**FIGURE 3:**
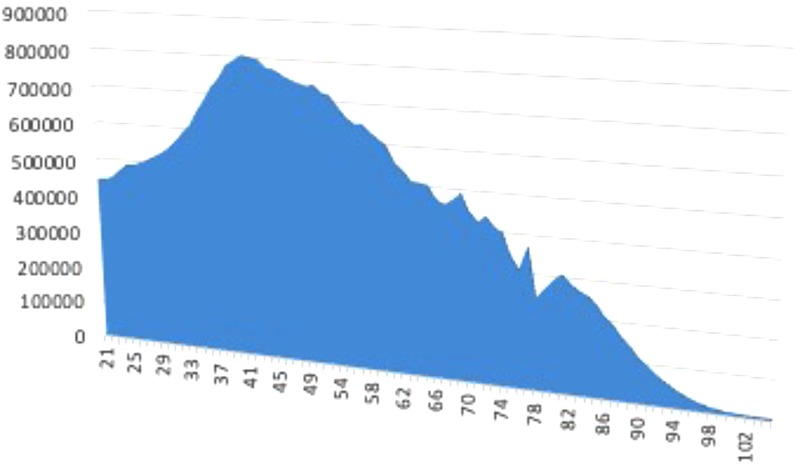
Spanish age distribution pyramid, population over the age of 21.

The breakdown of net monthly household income for the sample was as follows: 6.2% less than €1,000; 13.2% from €1,001 to €1,749; 13.9% from €1,750 to €2,499; 16.4% from €2,500 to €2,999; 38.3% more than €3,000; and 11.9% offering no response. As can be seen, income levels were quite high, which is reasonable given that the sample consisted of college-educated adults, who are more likely to earn higher salaries. This distribution is similar to that of the Spanish population as a whole. According to the Spanish National Institute of Statistics (Instituto Nacional de Estadistica [INE], 2017), 49.3% of people with a university degree earn salaries categorized in the highest level.

### Statistical Methodology

The research used the following sequential statistical process:

• Stage 1. Measurement model analysis
  Principal component exploratory factor analysis with Varimax rotation was performed to check for the possible existence of dimensions in the scales. Reliability and convergent and discriminant validity analyses of the scales were then performed. The removal of items from the scales based on these analyses was decided at this stage.
• Stage 2. Explanatory model of the intention to use cryptocurrencies (analysis of the structural model)
  • We analyzed the proposed explanatory model for the intention to use cryptocurrencies, calculating R^2^, Q^2^, path coefficients, and their estimated degree of significance. The analysis was done with consistent partial least squares structural equation modeling (PLSc-SEM). Dijkstra and Henseler (2015) established that PLSc-SEM is less sensitive to Type I and Type II errors than PLS-SEM. This analysis is also recommended when data do not follow a normal distribution or it is uncertain that they do. We ruled out PLS-MES because that method tends to skew factor loadings upward and underestimate regression coefficients (Gefen et al., 2011). PLSc-SEM can be used with models in which all constructs are reflective, as in the case at hand.

## Results

The intention to use cryptocurrencies was low. The arithmetic mean of the intention to use them was a 3 on a scale of 10. When respondents were asked about their use in the near future, the score increased to an average of 4, very close to the breaking point between using or not using cryptocurrencies (5). Standard deviations were high (the coefficient of variation was 1.08 for the intention to use and 0.83 for predicted use). Given the dispersion in the intention to use, it was highly advisable to develop an explanatory model to understand cryptocurrency acceptance behaviors. With this aim, we proposed the aforementioned model based on variables accepted by the scientific and academic community with high explanatory power regarding variability in the intention to use new technologies and products.

### Analysis of the Measurement Model

We performed an exploratory factor analysis to test the number of dimensions included in each scale. Each scale was found to have only one dimension. For all the scales, the Bartlett’s test of sphericity coefficient had a significance level less than 0.00, the Kaiser-Meyer-Olkin (KMO) statistic, which measures sampling adequacy, was greater than or equal to 0.5 (for two items the KMO was always = 0.5), and the percentage of variance explained by the factors was higher than 70%, which confirms the correct statistical functioning. From an exploratory perspective, it was confirmed that the scales did not include any mental structures with more than one dimension.

Regarding the evaluation of the measurement mode, according to Hair et al. (2011, 2013), in order to obtain a correct reliability indicator in reflective measurement models, the standardized loadings of the variables should be greater than 0.7 and significant (value *t* > 1.96) (Table 2). One of the observed variables showed a standardized loading slightly less than 0.7, but *t*-values greater than 1.96. In that case, we kept the variable based on Chin (1998) because the standardized loading rule of 0.7 is flexible, particularly when the indicators contribute to the validity of the factor content.

**Table 2:** Standardized loadings and *t*-values.

Construct/item	Loading
	(*t*-value)
**Intention to use**	
I intend to use cryptocurrencies	0.90 (52.16)
I predict that I will use cryptocurrencies	0.91 (48.22)
**Performance expectancy**	
Using cryptocurrencies will increase my opportunities to achieve important goals for me	0.97 (69.60)
Using cryptocurrencies will help me achieve my goals more quickly	0.93 (69.35)
Using cryptocurrencies will increase my standard of living	0.92 (55.40)
**Effort expectancy**	
It will be easy for me to learn to use cryptocurrencies	0.89 (38.66)
Using cryptocurrencies will be clear and understandable for me	0.95 (58.16)
It will be easy for me to use cryptocurrencies	0.94 (62.97)
It will be easy for me to become an expert in the use of cryptocurrencies	0.94 (49.45)
**Social influence**	
The people who are important to me will think that I should use cryptocurrencies	0.91 (43.21)
The people who influence me will think that I should use cryptocurrencies	0.93 (48.28)
People whose opinions I value would like me to use cryptocurrencies	0.99 (70.56)
**Facilitating conditions**	
I have the necessary resources to use cryptocurrencies	0.79 (23.27)
I have the necessary knowledge to use cryptocurrencies	0.88 (32.08)
Cryptocurrencies are compatible with other technologies that I use	0.78 (21.61)
I can get help if I have difficulty using cryptocurrencies	0.77 (20.66)
**Perceived risk**	
Using cryptocurrencies is risky	0.90 (6.30)
There is too much uncertainty associated with the use of cryptocurrencies	0.65 (5.98)
Compared with other currencies/investments, cryptocurrencies are riskier	0.87 (6.65)
**Financial literacy**	
I have a good level of financial knowledge	1.00 (62.58)
I have a high capacity to deal with financial matters	0.92 (33.56)

All constructs had a composite reliability and Cronbach’s alpha greater than 0.7, confirming that the construct reliability was adequate (see Table 3). The scales also showed an average variance extracted (AVE) greater than or equal to 0.5; the convergent validity criterion was thus met. The HTMT values were correct in all cases (<0.9) (Gold et al., 2001), and the square root of the AVE was greater than the correlations between constructs, proving that the discriminant validity criterion was also met (Roldán and Sánchez-Franco, 2012) (Table 4).

**Table 3:** Construct reliability (composite reliability and Cronbach’s alpha) and convergent validity (AVE).

Construct	Composite	Cronbach’s	AVE
	reliability	alpha	
Intention to use (IU)	0.898	0.897	0.814
Performance expectancy (PE)	0.960	0.960	0.889
Effort expectancy (EE)	0.962	0.962	0.864
Social influence (SI)	0.959	0.959	0.887
Facilitating conditions (FCs)	0.878	0.878	0.645
Perceived risk (PR)	0.850	0.851	0.658
Financial literacy (FL)	0.956	0.955	0.916

**Table 4:** Divergent validity.

Construct	IU	PE	EE	SI	FC	PR	FL
Intention to use (IU)	**0.902**	0.896	0.640	0.680	0.674	0.120	0.282
Performance expectancy (PE)	0.896	**0.943**	0.557	0.739	0.565	0.137	0.237
Effort expectancy (EE)	0.640	0.557	**0.930**	0.493	0.767	0.088	0.450
Social influence (SI)	0.680	0.739	0.494	**0.942**	0.566	0.089	0.239
Facilitating conditions (FCs)	0.673	0.565	0.767	0.563	**0.803**	0.094	0.489
Perceived risk (PR)	-0.123	-0.137	-0.090	-0.084	0.047	**0.817**	0.284
Financial literacy (FL)	0.282	0.237	0.450	0.239	0.493	0.286	**0.957**

*Bold data on the diagonal are the square root of the AVE. Data located below the diagonal are the correlations between the constructs. Data above the diagonal are the HTMT values.*

### Explanatory Model of the Intention to Use Cryptocurrencies (Structural Model Analysis)

**FIGURE 4:**
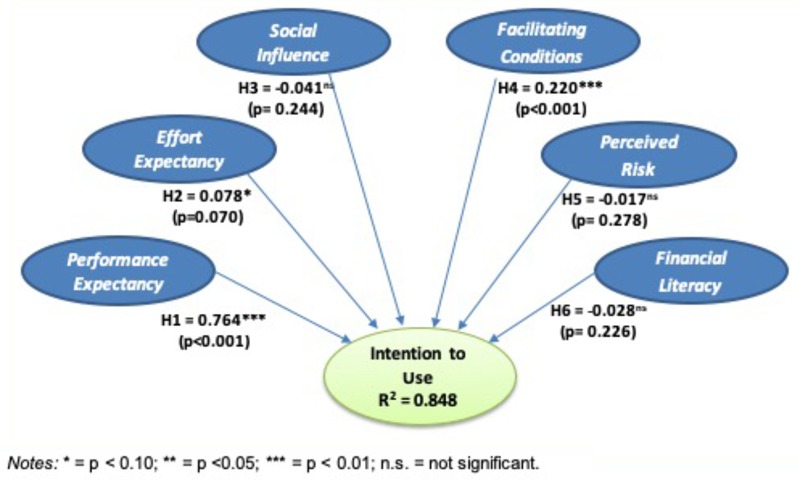
Graphical model of the influence of the explanatory variables (path coefficients) on the intention to use cryptocurrencies and R^2^.

Consistent PLS bootstrapping with 5000 resamples was used to evaluate the relevance of the path coefficients. Figure 4 shows the model’s overall results: R^2^ for the dependent variable and the path coefficients of the explanatory variables.

The model’s goodness of fit is very high, as can be seen in Table 5. *R*^2^ = 0.848, meaning that the model’s explanatory power is very high, since the explanatory variables explain 84.8% of the variance in the intention to use cryptocurrencies. Regarding the predictive power of the model, we used the Q^2^ provided by PLS predict (Shmueli et al., 2016). The Q^2^ obtained with PLS predict was greater than 0, and Q^2^ values greater than zero indicate that the exogenous constructs have predictive relevance. It is thus confirmed that the model strongly explains the intention to use cryptocurrencies. The average variance explained by each antecedent variable of the intention to use is shown in Table 5. As can be seen, this value was negative in some cases “due to the fact that the original relationship between the two variables is so close to zero that the difference in the signs simply reflects random variation around zero” (Falk and Miller, 1992, p. 75).

**Table 5:** Goodness of fit of the model, direct effects, *p*-value, correlation with the dependent variable and variance explained by the explanatory variables.

	R^2^	Q^2^	Direct effect	*p*-value	Correlation	Variance explained
Intention to use (IU)	0.848	0.654				
Performance expectancy (PE)			0.764	0.000	0.896	68.45%
Effort expectancy (EE)			0.078	0.070	0.640	4.99%
Social influence (SI)			-0.041	0.244	0.680	-2.79%
Facilitating conditions (FCs)			0.220	0.000	0.673	14.81%
Perceived risk (PR)			-0.017	0.278	-0.123	0.21%
Financial literacy (FL)			-0.028	0.226	0.282	-0.79%

The results indicate that performance expectancy and facilitating conditions significantly influence the intention to use cryptocurrencies. Support was thus found for hypotheses H_1_ and H_4_. Effort expectancy (EE) also had a significant effect, but at the lowest level (sig = 0.07). Therefore, although support was also found for H_2_, this support was less clear. No support was found for the rest of the hypotheses (H_3_, H_5_, and H_6_).

With the objective of producing valid predictions of behavioral intention to use cryptocurrencies, we used PLS predict (Shmueli et al., 2016; Felipe et al., 2017). In general, if we compare the results of PLS (partial least squares) with LM (linear model), PLS predict allows predictions very close to those obtained by using LM (Table 6).

**Table 6:** Partial least square predict assessment.

	PLS			LM			PLS-LM		
	RMSE	MAE	Q^2^	RMSE	MAE	Q^2^	RMSE	MAE	Q^2^
IU_1_	1.91	1.37	0.66	1.92	1.41	0.65	-0.02	-0.04	0.01
IU_2_	1.94	1.42	0.66	1.99	1.44	0.65	-0.05	-0.02	0.02

*IU, behavioral intention; RMSE, root mean squared error; MAE, mean absolute error; PLSs, partial least squares; LM, linear model.*

## Discussion and Conclusion

This research sought to test an explanatory model of the intention to use a new financial technology, namely, blockchain-based cryptocurrencies. The proposed model was based on variables from UTAUT technology acceptance models. Perceived risk and financial literacy were also added, as variables specifically used in the analysis of fintech acceptance. The proposed model explains 84.8% of the variance in the intention to use.

The results indicate that the variables with the greatest explanatory power for an individual investor’s intention to use cryptocurrencies are performance expectancy (explained 68.45% of the variance in the intention to use) and facilitating conditions (14.81%). Effort expectancy also had significant explanatory power, but the influence was smaller (4.99%). The remaining variables (social influence, perceived risk, and financial literacy) did not have a significant influence (*p*-value > 0.1).

The high explanatory power of performance expectancy gives rise to the first finding: performance expectancy is the determinant variable in the acceptance of cryptocurrency financial technologies. This finding is consistent with other studies that have found this variable to be determinant in the intention to use a given financial technology, including a biometric payment service (Kim et al., 2018), plastic money (Makanyeza and Mutambayashata, 2018), online banking (Khan et al., 2017; Sánchez-Torres et al., 2018), and m-banking (Kishore and Sequeira, 2016; Nisha, 2016; Farah et al., 2018; Hussain et al., 2018; Warsame and Ireri, 2018). Studies about cryptocurrencies and bitcoin in particular have reached the same results regarding the influence of performance expectancy on the intention to use, including in relation to electronic payments with cryptocurrencies (Mendoza-Tello et al., 2018) and bitcoin acceptance in China (Shahzad et al., 2018). Perceived usefulness is also the most significant variable influencing the intention to use bitcoin (Walton and Johnston, 2018).

The variable with the second highest explanatory power was facilitating conditions. There is no consensus regarding the influence of facilitating conditions on the acceptance of financial technologies. Several studies have confirmed its influence (Khan et al., 2017; Hussain et al., 2018), while others have found no evidence that it influences fintech acceptance (Farah et al., 2018; Makanyeza and Mutambayashata, 2018; Moon and Hwang, 2018).

With regard to effort expectancy, most of the literature suggests that it does influence financial technology acceptance (e.g., Kishore and Sequeira, 2016; Nisha, 2016; Farah et al., 2018; Hussain et al., 2018; Kim et al., 2018; Makanyeza and Mutambayashata, 2018; Moon and Hwang, 2018; Sánchez-Torres et al., 2018). However, some authors have shown that effort expectancy does not influence fintech acceptance (Khan et al., 2017) or does not influence the intention to use it equally in all segments (Warsame and Ireri, 2018). As for findings regarding cryptocurrency fintech in particular, effort expectancy has been shown to have a positive influence on cryptocurrency adoption (Schaupp and Festa, 2018) and on bitcoin acceptance in China (Shahzad et al., 2018). Our results support the mainstream findings regarding the influence of effort expectancy on fintech acceptance: it is a significant factor. However, it is not the most influential one, nor is it critical to successful cryptocurrency acceptance compared to performance expectancy and facilitating conditions. A bitcoin study in South Africa (Walton and Johnston, 2018) yielded similar findings.

Various factors should be considered in relation to the analyses of the variables that were not statistically significant. Given the current early stages of the development of cryptocurrency financial technologies and their technological basis (blockchain), it might initially seem surprising that perceived risk was not found to be relevant to their adoption. Because of the anonymity (pseudonymity) and elimination of trusted intermediaries that cryptocurrencies entail, they can potentially be used for criminal activities (e.g., money laundering, illicit marketplaces, and ransomware) (Juels et al., 2016). The reason for the present finding is the low variability of the explanatory variable (perceived risk), which does not explain the variability in the intention to use cryptocurrencies. However, that does not mean that it is not an important factor in cryptocurrency acceptance. Support for this argument can be found in other industries. For instance, in the hotel industry, the degree of cleanliness of a high-end hotel has no explanatory power with regard to hotel choice, because customers in general assume that a high-end hotel will be clean. This results in very low variability in the variable, such that cleanliness is not an influential variable in hotel choice. Thus, a very important variable (cleanliness) can play a critical role (its absence would have a strong negative impact on the evaluation of the service), yet not be determinant in high-end hotel expectations and choice (Medrano et al., 2016). This same logic can be applied to the cryptocurrency acceptance decision. Thus, the arithmetic mean of the three observable variables measuring perceived risk is 7.3 (on a scale of 0 to 10), indicating that the perceived risk is quite high; however, its variability (coefficient of variation 0.36) is insufficient to explain the intention to use cryptocurrencies (with a coefficient of variation of 1.08 and 0.83 for the scale’s three observable variables). That means that despite being a critical factor in cryptocurrency acceptance, risk does not affect the intention to use cryptocurrencies because most people assume that operating with them is risky. Shaikh et al. (2018) report similar findings, noting that perceived risk is not a determinant variable in the intention to use m-banking technologies, but is critical in the preadoption process. Farah et al. (2018) and Moon and Hwang (2018) find that perceived risk does not explain the decision to use a new financial technology, which is also consistent with our findings. In their study specifically of cryptocurrencies, Mendoza-Tello et al. (2018) show that perceived risk is not a significant factor in explaining the intention to use cryptocurrencies for electronic payments. Likewise, Walton and Johnston (2018) show that the perceived security risk does not influence attitude toward or the intention to use bitcoin.

Another finding of our research is the non-significant role of social influence in explaining the intention to use cryptocurrencies. Previous studies reached the same conclusion: this variable does not influence the adoption of other financial technologies, such as plastic money (Makanyeza and Mutambayashata, 2018) and online banking (Khan et al., 2017). However, opposite findings have also been reported, as in the m-banking studies by Kishore and Sequeira (2016); Mahfuz et al. (2016), and Warsame and Ireri (2018) that have found social influence to be relevant to adoption. The results of cryptocurrency acceptance studies are similarly contradictory. A study on electronic payments with cryptocurrencies considered the influence of social norm on acceptance to be non-significant, while other studies have found it to be significant, including one study on cryptocurrency adoption (Schaupp and Festa, 2018) and another on bitcoin acceptance (Shahzad et al., 2018). With regard to cryptocurrency adoption, our findings indicate that social influence will not be key.

Finally, we found that financial literacy has no power as an explanatory variable for cryptocurrency acceptance. Other studies about financial literacy have found that people with greater financial knowledge are less likely to make little-reasoned investments (Lam and Lam, 2017). In that regard, Stolper and Walter (2017, p. 613) note that “a voluminous literature analyzes the question whether high levels of financial literacy trigger superior financial decision making. As we will review shortly, the majority of papers document a positive correlation between measures of financial literacy and sound financial behavior in various domains.” Based on our results, it cannot be demonstrated that greater financial knowledge influences the decision to use cryptocurrencies. This is because financial literacy allows people to make better financial decisions. In some cases, the best decision could be not to invest, while in another it might be to invest. Our results contribute to previous findings. Greater financial knowledge allows a customer to more accurately evaluate the investment (e.g., whether to invest in bitcoin or ethereum depending on the status of the financial market at any given time), but not the technology that supports it (cryptocurrency technologies in the present case). For this reason, financial literacy could influence the decision at the investment level, but does not have any significant influence on the decision at the technology level, which is the focus of the present research. Thus, from a financial literacy perspective, the decision of whether or not to use a cryptocurrency could be based on financial criteria, not technology acceptance ones.

Based on our findings, we propose several measures to operate with a greater likelihood of success in the cryptocurrencies and blockchain-related services market. The first recommendation concerns the risk related to operating in these markets. The perceived risk of cryptocurrency transactions is very high; given the current status of the necessary technological development, customers and investors view investing in or operating with these new technological assets as very risky. Therefore, future cryptocurrencies should seek to solve that problem as a condition for pre-adoption. The firsts cryptocurrencies to be seen as “risk-free” could gain an important competitive advantage in relation to the current offer.

Second, the product and service design for a new cryptocurrency (or the innovation efforts for current ones) should focus on performance as the most critical adoption factor. Cryptocurrency must become a high-value-added proposition for customers, and considerable marketing efforts must be undertaken to ensure that potential customers perceive this value. The more value added offered by a cryptocurrency, the more likely it is to be used. Focusing on usefulness is a recommended strategy in the cryptocurrency market.

The third recommendation concerns facilitating conditions. The intention to use a current or new cryptocurrency is heavily dependent on the conditions under which potential customers can operate with them. Factors such as the technological resources and technical knowledge needed to operate with a cryptocurrency, the compatibility of a customer’s technology with cryptocurrency technical requirements, the existence of widely accepted standards for operating with them, or the existence of an easily accessed helpdesk in case of problems are all important factors that could affect cryptocurrency adoption.

The fourth recommendation has to do with the effort a customer needs to make to use a cryptocurrency. Even through the effort required to learn and operate with a cryptocurrency is not one of the most important factors for acceptance, it is significant. Any innovation in a cryptocurrency’s usability will thus positively influence the intention to use it.

Finally, this research has some limitations. We focus on a very specific population segment: college-educated adults with a basic grasp of the Internet. Notwithstanding our discussion of and rationale for this decision, future studies should focus on other segments in order to gain a broader knowledge of cryptocurrency acceptance in society. Another possible limitation is that this research was circumscribed to Spain. The results might be different if the survey had had a larger geographical scope or been conducted in another country (e.g., the results of the aforementioned m-banking studies differed depending on the region, while the ING study (Exton and Doidge, 2018) revealed different perceptions depending on the country). Thus, future research should be conducted in other countries. Another factor that could be included in future research is sustainability of cryptocurrencies and blockchain mining. According to Krause and Tolaymat (2018) the mining process requires intensive computation resources with large energy consumption, being estimated than during the period from 2016 to 2018 the energy needed to mine 1 US$ of Bitcoins was 17 megajoules compared with the 5 megajoules needed to obtain 1US$ of gold. Based on this finding, sustainability factors could have an implact on cryptocurrencies development. Cryptocurrencies are an emerging technology in constant evolution. Therefore, the findings of the present research should be interpreted with care. In the near future, technology will continue to change, as will people’s knowledge of financial technology. Consequently, future research should both include a longitudinal study to track the evolutionary adoption of cryptocurrencies and seek to update the model to future circumstances.

## Author Contributions

All authors listed have made a substantial, direct and intellectual contribution to the work, and approved it for publication.

## Conflict of Interest Statement

The authors declare that the research was conducted in the absence of any commercial or financial relationships that could be construed as a potential conflict of interest.

## References

[B1] Ajzen I. (1991). The theory of planned behavior. *Organ. Behav. Hum. Decis. Proces.* 50 179–211. 10.1016/0749-5978(91)90020-T

[B2] Bloomberg J. (2017). *Using Bitcoin or Other Cryptocurrency to Commit Crimes? Law Enforcement Is onto You. Forbes.* Available at: https://www.forbes.com/sites/jasonbloomberg/2017/12/28/using-bitcoin-or-other-cryptocurrency-to-commit-crimes-law-enforcement-is-onto-you/#1006bdfc3bdc

[B3] Bort J. (2014). *Is Bitcoin Pizza Day Thanks to These Two Pizzas Worth $5 Million Today. Business Insider, 22 May.* Available at: https://www.businessinsider.es/bitcoin-price-pizza-day-may-22-2018-5?r=US&IR=T

[B4] Business Wire (2017). *$16.3 Billion Global Blockchain Technology Market Analysis & Trends - Industry Forecast to 2025 - Research and Markets | Business Wire.* Available at: https://www.businesswire.com/news/home/20170130005684/en/16.3-Billion-Global-Blockchain-Technology-Market-Analysis

[B5] Carson B. Romanelli G. Walsh P. Zhumaev A. (2018). *Blockchain Beyond the Hype: What Is the Strategic Business Value? | McKinsey&Company. Digital McKinsey, (June).* Available at: https://www.mckinsey.com/business-functions/digital-mckinsey/our-insights/blockchain-beyond-the-hype-what-is-the-strategic-business-value

[B6] CCN (2016). *Here’s Why Bitcoin Is Going Nowhere Fast: Financial Literacy.* Available at: https://www.ccn.com/bitcoin-financial-literacy/

[B7] Chin W. W. (1998). Issues and opinion on structural equation modelling. *MIS Q.* 22 7–15.

[B8] Coin Market Cap (2018). *All Cryptocurrencies | CoinMarketCap.* Available at: https://coinmarketcap.com/all/views/all/.

[B9] Coinmap (2018). *Coinmap.* Available at: https://coinmap.org/welcome/.

[B10] Davis F. D. (1989). Perceived usefulness, perceived ease of use, and user acceptance of information technology. *MIS Q.* 13 319–340. 10.2307/249008

[B11] Deloitte (2015). *State-Sponsored Cryptocurrency: Adapting the Best of Bitcoin’s Innovation to the Payments Ecosystem.* Available at: https://www2.deloitte.com/content/dam/Deloitte/au/Documents/financial-services/deloitte-au-fs-state-sponsored-cryptocurrency-180516.pdf.

[B12] Dijkstra T. K. Henseler J. (2015). Consistent Partial Least Squares Path Modeling. *MIS Q.* 39 297–316. 10.25300/MISQ/2015/39.2.02

[B13] Exton J. Doidge F. (2018). *Cracking the Code on Cryptocurrency - ING Bank. ING International Survey Mobile Banking* Available at: https://think.ing.com/reports/cracking-the-code-on-cryptocurrency/

[B14] Falk R. F. Miller N. B. (1992). *A Primer for Soft Modeling.* Akron, OH: University of Akron Press.

[B15] Faqih K. M. S. (2016). An empirical analysis of factors predicting the behavioral intention to adopt Internet shopping technology among non-shoppers in a developing country context: does gender matter? *J. Retailing Consum. Serv.* 30 140–164. 10.1016/j.jretconser.2016.01.016

[B16] Farah M. F. Hasni M. J. S. Abbas A. K. (2018). Mobile-banking adoption: empirical evidence from the banking sector in Pakistan. *Int. J. Bank Mark.* 36 1386–1413. 10.1108/IJBM-10-2017-0215

[B17] Featherman M. S. Pavlou P. A. (2003). Predicting e-services adoption: a perceived risk facets perspective, International. *J. Hum. Comput. Stud.* 59 451–474. 10.1016/S1071-5819(03)00111-3

[B18] Federal Reserve System (2017). *Federal Reserve Next Steps in the Payments Improvement Journey.* Available at: https://www.federalreserve.gov/newsevents/pressreleases/files/other20170906a1.pdf

[B19] Felipe C. M. Roldán J. L. Leal-Rodríguez A. L. (2017). Impact of organizational culture values on organizational agility. *Sustainability* 9:2354 10.3390/su9122354

[B20] Fishbein M. Ajzen I. (1975). *Belief, Attitude, Intention, and Behavior: An Introduction to Theory and Research.* Boston, MA: Addison-Wesley.

[B21] Gao X. Clark G. D. Lindqvist J. (2016). “Of Two Minds, Multiple Addresses, and One Ledger,” in *Proceedings of the 2016 CHI Conference on Human Factors in Computing Systems - CHI ’16* (San Jose, CA) 1656–1668. 10.1145/2858036.2858049

[B22] Gefen D. Rigdon E. E. Straub D. W. (2011). An update and extension to SEM guidelines for administrative and social science research. *MIS Q.* 35 3–14. 10.2307/23044042

[B23] Gold A. H. Malhotra A. Segars A. H. (2001). Knowledge management: an organizational capabilities perspective. *J. Manag. Inf. Syst.* 18 185–214. 10.1080/07421222.2001.11045669

[B24] Hair J. F. Ringle C. M. Sarstedt M. (2011). PLS-SEM: indeed a silver bullet. *J. Mark. Theory Pract.* 19 139–151. 10.2753/MTP1069-6679190202

[B25] Hair J. F. Ringle C. M. Sarstedt M. (2013). Partial least squares structural equation modeling: rigorous applications better result and higher acceptance. *Long Range Plan.* 46 1–12. 10.1016/j.lrp.2013.01.001

[B26] Hastings J. S. Madrian B. C. Skimmyhorn B. (2013). Financial literacy, financial education, and economic outcomes. *Annu. Rev. Econ.* 5 347–375. 10.1146/annurev-economics-082312-125807 23991248 PMC3753821

[B27] Holub M. Johnson J. (2018). Bitcoin research across disciplines. *Inf. Soc.* 34 114–126. 10.1080/01972243.2017.1414094

[B28] Hussain M. Mollik A. T. Johns R. Rahman M. S. (2018). M-payment adoption for bottom of pyramid segment: an empirical investigation. *Int. J. Bank Mark.* 37 362–381. 10.1108/IJBM-01-2018-0013

[B29] ICObench (2018). *ICO Market Reports | ICObench.* Available at: https://icobench.com/report?utm_campaign=im2018report&utm_source=statsandfacts.

[B30] Instituto Nacional de Estadistica [INE] (2017). *Decil de Salarios Del Empleo Principal. Encuesta de Población Activa (EPA), Año 2016.* Available at: http://www.ine.es/prensa/epa_2016_d.pdf

[B31] Juels A. Kosba A. Shi E. (2016). “The ring of gyges,” in *Proceedings of the 2016 ACM SIGSAC Conference on Computer and Communications Security - CCS’16* (New York, NY) 283–295. 10.1145/2976749.2978362

[B32] Kannungo S. Jain V. (2004). “Relationship between risk and intention to purchase in an online context: role of gender and product category,” in *Proceedings of the 13th European Conference on Information Systems, The European IS Profession in the Global Networking Environment, ECIS 2004* (Turku).

[B33] Khan I. U. Hameed Z. Khan S. U. (2017). Understanding online banking adoption in a developing country: UTAUT2 with cultural moderators. *J. Glob. Inf. Manag.* 25 43–65. 10.4018/JGIM.2017010103

[B34] Kim S. Y. Lee S. H. Chi Y. D. Im E. T. Gim G. Y. (2018). A study on the factors affecting the intention to use biometrics in payment services. *Int. J. Bank Mark.* 36 170–183.

[B35] Kishore S. K. Sequeira A. H. (2016). An empirical investigation on mobile banking service adoption in rural Karnataka. *SAGE Open* 6 2158244016633731 10.1177/2158244016633731

[B36] Krause M. J. Tolaymat T. (2018). Quantification of energy and carbon costs for mining cryptocurrencies. *Nat. Sustain.* 1 711–718. 10.1038/s41893-018-0152-7

[B37] Krombholz K. Judmayer A. Gusenbauer M. Weippl E. (2017). “The other side of the coin: user experiences with bitcoin security and privacy,” in *Financial Cryptography and Data Security* Vol. 9603 eds Grossklags J. Preneel B. Hutchison D. 555–580.

[B38] Lam L. T. Lam M. K. (2017). The association between financial literacy and problematic internet shopping in a multinational sample. *Addict. Behav. Rep.* 6 123–127. 10.1016/j.abrep.2017.10.002 29450247 PMC5800552

[B39] Lin J. T. Lusardi A. Mottola G. R. Kieffer C. Walsh G. (2016). *Financial Capability in the United States 2016.* Available at: http://www.usfinancialcapability.org/downloads/NFCS_2015_Report_Natl_Findings.pdf

[B40] López de la Cruz L. (2002). La presencia de la mujer en la universidad española. *Rev. Hist. Educ. Latinoam.* 4 291–299.

[B41] Lusardi A. Mitchell O. S. (2014). The economic importance of financial literacy: theory and evidence. *J. Econ. Lit.* 52 5–44. 10.1257/jel.52.1.5 28579637 PMC5450829

[B42] Mahfuz M. A. Khanam L. Mutharasu S. A. (2016). “The influence of website quality on m-banking services adoption in bangladesh: applying the UTAUT2 model using PLS,” in *Proceedings of the 2016 International Conference on Electrical, Electronics, and Optimization Techniques (ICEEOT)* (Piscataway, NJ: IEEE) 2329–2335. 10.1109/ICEEOT.2016.7755110

[B43] Makanyeza C. Mutambayashata S. (2018). Consumers’ acceptance and use of plastic money in Harare, Zimbabwe: application of the unified theory of acceptance and use of technology 2. *Int. J. Bank Mark.* 36 379–392. 10.1108/IJBM-03-2017-0044

[B44] Medrano N. Olarte-Pascual C. Pelegrín-Borondo J. Sierra-Murillo Y. (2016). Consumer behavior in shopping streets: the importance of the salesperson’s professional personal attention. *Front. Psychol.* 7:125. 10.3389/fpsyg.2016.00125 26903927 PMC4748058

[B45] Mendoza-Tello J. C. Mora H. Pujol-Lopez F. A. Lytras M. D. (2018). Social commerce as a driver to enhance trust and intention to use cryptocurrencies for electronic payments. *IEEE Access* 6 50737–50751. 10.1109/ACCESS.2018.2869359

[B46] Moon Y. Hwang J. (2018). Crowdfunding as an alternative means for funding sustainable appropriate technology: acceptance determinants of backers. *Sustainability* 10:1456 10.3390/su10051456

[B47] Morris D. Z. (2018). *Nearly Half of 2017’s Bitcoin-Backed «ICO» Projects Have Collapsed | Fortune.* Available at: http://fortune.com/2018/02/25/cryptocurrency-ico-collapse/

[B48] Nakamoto S. (2008a). *Bitcoin P2P E-cash Paper.* Available at: https://www.mail-archive.com/cryptography@metzdowd.com/msg09959.html

[B49] Nakamoto S. (2008b). *Bitcoin: A Peer-to-Peer Electronic Cash System.* Available at: https://bitcoin.org/bitcoin.pdf.

[B50] Natarajan H. Krause S. K. Gradstein H. L. (2017). *Distributed Ledger Technology (DLT) and Blockchain. World Bank Group.* Available at: http://documents.worldbank.org/curated/en/177911513714062215/Distributed-Ledger-Technology-DLT-and-blockchain. 10.1596/29053

[B51] Nisha N. (2016). Exploring the dimensions of mobile banking service quality: implications for the banking sector. *Int. J. Bus. Anal.* 3 60–76. 10.4018/IJBAN.2016070104

[B52] Pelegrín-Borondo J. Arias-Oliva M. González-Menorca L. Juaneda-Ayensa E. (2015). Pricing policies in hotels: a psychological threshold research in online and offline channels. *Int. J. Internet Mark. Advert.* 9 161–179. 10.1504/IJIMA.2015.070720

[B53] Roldán J. L. Sánchez-Franco M. J. (2012). “Variance based structural equation modeling: guidelines for using partial least squares in information systems research,” in *Research Methodologies, Innovations and Philosophies in Software Systems Engineering and Information Systems* eds Mora M. Gelman O. Steenkamp A. Raisinghani M. (Hershey, PA: Raisinghan Information Science Reference) 193–222. 10.4018/978-1-4666-0179-6.ch010

[B54] Salisbury W. D. Pearson R. A. Pearson A. W. Miller D. W. (2001). Perceived security and world wide web purchase intention. *Ind. Manag. Data Syst.* 101 165–176. 10.1108/02635570110390071 27516749

[B55] Sánchez-Torres J. A. Canada F. J. A. Sandoval A. V. Alzate J. A. S. (2018). E-banking in Colombia: factors favouring its acceptance, online trust and government support. *Int. J. Bank Mark.* 36 170–183. 10.1108/IJBM-10-2016-0145

[B56] Schatsky D. Muraskin C. (2015). *Beyond bitcoin: Blockchain is coming to disrupt your industry. Deloitte University Press.* Available at: https://www2.deloitte.com/content/dam/insights/us/articles/trends-blockchain-bitcoin-security-transparency/DUP_1381_Beyond-bitcoin_SFS_vFINAL.pdf.

[B57] Schaupp L. C. Festa M. (2018). “Cryptocurrency Adopt ion and the Road to Regulation,” in *Proceedings of the 19th Annual International Conference on Digital Government Research: Governance in the Data Age* ed. Zuiderwijk A. C. C. H. (Delft: ACM) 1–9.

[B58] Shahzad F. Xiu G. Wang J. Shahbaz M. (2018). An empirical investigation on the adoption of cryptocurrencies among the people of mainland China. *Technol. Soc.* 55 33–44. 10.1016/j.techsoc.2018.05.006

[B59] Shaikh A. A. Glavee-Geo R. Karjaluoto H. (2018). How relevant are risk perceptions, effort, and performance expectancy in mobile banking adoption? *Int. J. E Bus. Res.* 14 39–60. 10.4018/IJEBR.2018040103

[B60] Shim S. I. Lee Y. (2011). Consumer’s perceived risk reduction by 3D virtual model. *Int. J. Retail Distrib. Manag.* 39 945–959. 10.1108/09590551111183326

[B61] Shmueli G. Ray S. Estrada J. M. V. Chatla S. B. (2016). The elephant in the room: predictive performance of PLS models. *J. Bus. Res.* 69 4552–4564. 10.1016/j.jbusres.2016.03.049

[B62] Simonite T. (2011). *What Bitcoin Is, and Why It Matters - MIT Technology Review. MIT Technology Review.* Available at: https://www.technologyreview.com/s/424091/what-bitcoin-is-and-why-it-matters/

[B63] Stolper O. A. Walter A. (2017). Financial literacy, financial advice, and financial behavior. *J. Bus. Econ.* 87 581–643. 10.1007/s11573-017-0853-9

[B64] Turner A. Samantha A. Irwin M. Turner A. Samantha A. Irwin M. (2018). Bitcoin transactions?: a digital discovery of illicit activity on the blockchain. *J. Financ. Crime* 25 109–130. 10.1108/JFC-12-2016-0078

[B65] Usebitcoins (2018). *useBitcoins.info.* Available at: https://usebitcoins.info/.

[B66] Van Rooij M. Lusardi A. Alessie R. (2011). Financial literacy and stock market participation. *J. Financ. Econ.* 101 449–472. 10.1016/j.jfineco.2011.03.006

[B67] Venkatesh V. Davis F. D. (2000). A theoretical extension of the technology acceptance model: four longitudinal field studies. *Manag. Sci.* 46 186–204. 10.1287/mnsc.46.2.186.11926

[B68] Venkatesh V. Morris M. G. Davis G. B. Davis F. D. (2003). User acceptance of information technology: toward a unified view. *MIS Q.* 27 425–478. 10.2307/30036540

[B69] Venkatesh V. Thong J. Xu X. (2012). Consumer acceptance and use of information technology: extending the unified theory of acceptance and use of technology. *MIS Q.* 36 157–178. 10.2307/41410412

[B70] Walton A. Johnston K. (2018). Exploring perceptions of bitcoin adoption: the South African virtual community perspective. *Interdiscip. J. Inf. Knowl. Manag.* 13 165–182.

[B71] Warsame M. H. Ireri E. M. (2018). Moderation effect on mobile microfinance services in Kenya: an extended UTAUT model. *J. Behav. Exp. Finance* 18 67–75. 10.1016/j.jbef.2018.01.008

[B72] World Economic Forum (2015). *WEF: Technology Tipping Points and Societal Impact.* Available at: http://www3.weforum.org/docs/WEF_GAC15_Technological_Tipping_Points_report_2015.pdf

